# Environmental assessment of Al-Hammar Marsh, Southern Iraq

**DOI:** 10.1016/j.heliyon.2017.e00256

**Published:** 2017-02-23

**Authors:** Hind Fadhil Abdullah Al-Gburi, Balsam Salim Al-Tawash, Hadi Salim Al-Lafta

**Affiliations:** Department of Geology, College of Science, University of Baghdad, Baghdad, Iraq

**Keywords:** Environmental science, Geochemistry, Biogeochemistry

## Abstract

**Aim:**

(a) To determine the spatial distributions and levels of major and minor elements, as well as heavy metals, in water, sediment, and biota (plant and fish) in Al-Hammar Marsh, southern Iraq, and ultimately to supply more comprehensive information for policy-makers to manage the contaminants input into the marsh so that their concentrations do not reach toxic levels. (b) to characterize the seasonal changes in the marsh surface water quality. (c) to address the potential environmental risk of these elements by comparison with the historical levels and global quality guidelines (i.e., World Health Organization (WHO) standard limits). (d) to define the sources of these elements (i.e., natural and/or anthropogenic) using combined multivariate statistical techniques such as Principal Component Analysis (PCA) and Agglomerative Hierarchical Cluster Analysis (AHCA) along with pollution analysis (i.e., enrichment factor analysis)

**Methods:**

Water, sediment, plant, and fish samples were collected from the marsh, and analyzed for major and minor ions, as well as heavy metals, and then compared to historical levels and global quality guidelines (WHO guidelines). Then, multivariate statistical techniques, such as PCA and AHCA, were used to determine the element sourcing.

**Results:**

Water analyses revealed unacceptable values for almost all physio-chemical and biological properties, according to WHO standard limits for drinking water. Almost all major ions and heavy metal concentrations in water showed a distinct decreasing trend at the marsh outlet station compared to other stations. In general, major and minor ions, as well as heavy metals exhibit higher concentrations in winter than in summer. Sediment analyses using multivariate statistical techniques revealed that Mg, Fe, S, P, V, Zn, As, Se, Mo, Co, Ni, Cu, Sr, Br, Cd, Ca, N, Mn, Cr, and Pb were derived from anthropogenic sources, while Al, Si, Ti, K, and Zr were primarily derived from natural sources. Enrichment factor analysis gave results compatible with multivariate statistical techniques findings. Analysis of heavy metals in plant samples revealed that there is no pollution in plants in Al-Hammar Marsh. However, the concentrations of heavy metals in fish samples showed that all samples were contaminated by Pb, Mn, and Ni, while some samples were contaminated by Pb, Mn, and Ni.

**Discussion and conclusions:**

Decreasing of Tigris and Euphrates discharges during the past decades due to drought conditions and upstream damming, as well as the increasing stress of wastewater effluents from anthropogenic activities, led to degradation of the downstream Al-Hammar Marsh water quality in terms of physical, chemical, and biological properties. As such properties were found to consistently exceed the historical and global quality objectives. However, element concentration decreasing trend at the marsh outlet station compared to other stations indicate that the marsh plays an important role as a natural filtration and bioremediation system. Higher element concentrations in winter were due to runoff from the washing of the surrounding Sabkha during flooding by winter rainstorms. Finally, the high concentrations of heavy metals in fish samples can be attributed to bioaccumulation and biomagnification processes.

## 1 Introduction

Wetlands are among the most productive ecosystems on Earth [1], and provide many important services to human society [2]. A rare aquatic landscape in a desert milieu, the Mesopotamian marshlands (hereafter “the Marshes”) is home to ancient communities rooted in the dawn of human history [3]. These marshes were once the largest wetlands in Southwest Asia and covered more than 15,000 km^2^ [4]. Originally covering considerable parts of the Mesopotamian Plain, which developed along the Euphrates and Tigris Rivers [5], Iraqi marshes are important as they have economic, social, and biodiversity value. They support coastal fisheries, which endows them with a truly global dimension, and they represent a permanent habitat for many unique species of plants, fish, invertebrate, and birds, and a flyway for millions of birds migrating between Siberia and Africa [6]. The Marshes and their inhabitants have witnessed three wars and were subjected to drying operations from the early 1980s, involving massive drainage works and upstream damming, and as a result were almost totally dry by 1991 [7, 8]. These drying operations have resulted in drastic changes in the marshes environment, which are still suffered today (e.g., creeping of the sand dunes towards ex-marsh areas, dryness of the land, increasing of Sabkha, degradation of flora and fauna, and migration of the local people) [9]. After 2003 the marshes were refilled but the degradation in water quality and ecosystem still endures.

Al-Hammar Marsh, one of the three biggest marshes in Iraq, is situated to the south of the Euphrates River (30 45'–30° 59' N, 46° 25'–47° 15' E) and has an area ranging from 2800 km^2^ of contiguous permanent marsh to 4500 km^2^ during flooding periods. The marsh that is fed mainly by the Euphrates River, Tigris River, the Central Marshes, and groundwater recharge drains ultimately into the Shatt Al-Arab River, which empties into the Arabian Gulf [10]. Al-Hammar Marsh had been desiccated for more than a decade; however, the marsh was restored to around half of its original size in 2005 after a policy was initiated to restore the marshes in 2003 [9].

While some studies have addressed the water quality and environmental status of Al-Hammar Marsh [4, 6, 11], few studies have applied new tools to investigate the possible sources of pollution and the impact of such pollution on aquatic life in the marsh. Therefore, the aim of the study is to address the distribution, levels, and sources of contaminants in both water and sediments in the marsh, in order to evaluate their environmental impacts and effects on the aquatic life, and, eventually, how to manage the contaminant input into the marsh so that their concentrations do not reach toxic levels. Furthermore, a joint initiative between the United Nations Environment Program (UNEP) and United Nations Educational, Scientific and Cultural Organization (UNESCO) has been established to ensure sustainable development of the Iraqi Marshes by introducing them into the World Heritage List, as these unique wetlands represent a region of outstanding universal historical, cultural, environmental, hydrological, and socio-economic value [12]. Therefore, we hope that the current study contributes to the management process, which not only meets the technical requirements of the World Heritage Convention, but will also give new impetus to efforts that aim to preserve the environment in the Mesopotamian Marshes.

## 2 Methods

### 2.1 Ethical clearance

Permission was obtained from the Iraqi Ministry of Environment prior to conducting the current study.

### 2.2 Sampling

#### 2.2.1 Water sampling

**Fig. 1:**
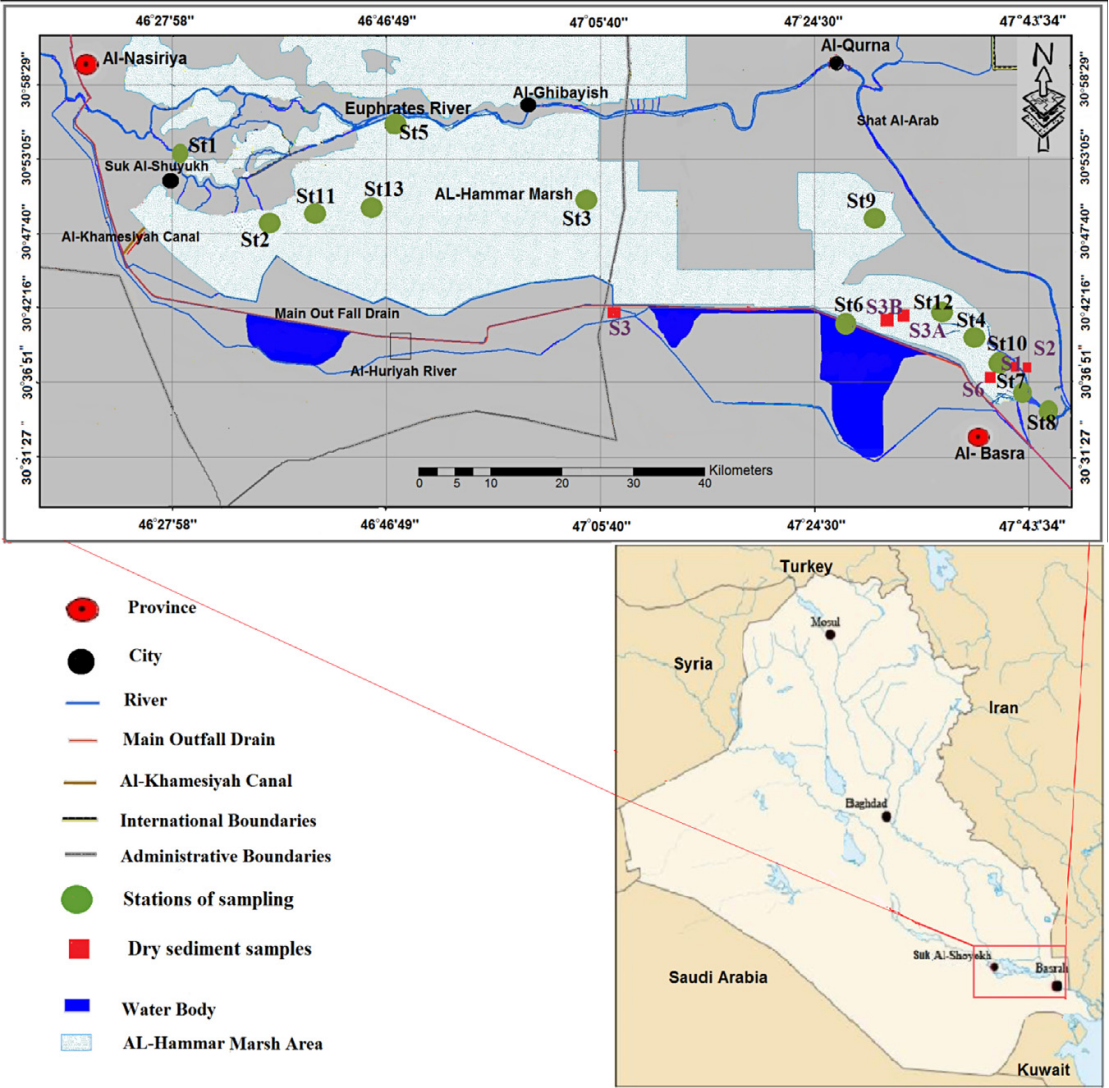
Map of field sampling stations in Al-Hammar Marsh.

The water samples were collected from Al-Hammar Marsh water during two seasons. Twelve water samples were taken from the marsh in January (represents winter season) 2014 and nine water samples in July (represents summer season) 2014 (Table 1 and Fig. 1).

#### 2.2.2 Sediments sampling

Seventeen samples from Al- Hammar Marsh sediments were collected during the winter season (Table 1 and Fig. 1).

#### 2.2.3 Plants and fish sampling

Twelve different plant samples of *Phragmites australis* (P2, P7, P8), *Typha domingensis* (P3, P4, P9), *Schoenoplectus litoralis* (P1, P6, P10), and *Ceratophyllum demersum* (P2, P5, P7) species were gathered from Al- Hammar Marsh from ten sampling stations (Table 1). The parts sampled from the plants were stems and leaves.

Fifteen fish samples from three fish species of *Liza abu* (F1, F3, F4, F5, F8), *Tilapia zilli* (F1, F2, F3, F5, F8), and *Carassius carassius* (F3, F4, F5, F8, F9) were gathered from seven sampling stations in the Al- Hammar Marsh (Table 1).

### 2.3 Sample analyses

Water depth, turbidity, Electrical Conductivity (EC), and Dissolved Oxygen (DO) of marsh water was measured in the field with a portable multimeter, which was previously calibrated, while the other physical and chemical characteristics of the water samples were analyzed in the lab according to the methods of the American Public Health Association (APHA) [13]. The gravimetric method [14], five −day Biological Oxygen Demand (BOD) test [15], and Colorimetric method [16] were used to determine Total Dissolved Solids (TDS), BOD, and NO_2_^−^, respectively. Ca^2+^, Mg^2+^, and Total Hardness (TH) were determined using Ethylenediaminetetraacetic acid (EDTA) method. Flame Photometry method was used to determine Na^+^ and K^+^ ions [17]. HCO_3_^−^ was determined via titration method using indicator titrated with HCl. SO_4_^2−^ was determined via the Turbidimetric method [18]. C1^−^ was determined via Silver Nitrate method [19]. NO_3_^−^ was determined via Ultraviolet Spectrophotometry method [20]. PO_4_^3−^ was determined via Ascorbic Acid method using a spectrophotometer.

Heavy metals in water samples were sent to the ALS Laboratory Group in north Vancouver, Canada to be analyzed by Inductively Coupled Plasma Mass Spectrometry (ICP-MS) type Agilent device. The samples were analyzed directly on the device without dilution and the result was corrected for any spectral interferences. Organic Matter percentage (OM%) in the sediment samples was determined by reducing the potassium dichromate (K_2_CrO_7_) by OC compound and subsequent determination of the unreduced dichromate by oxidation-reduction titration with ferrous ammonium sulfate [21]. Then, OM% was converted to percent total organic carbon (TOC%). Traditionally, for soils, a conversion factor of 1.724 is used to convert organic matter to organic carbon based on the assumption that organic matter contains 58% organic carbon (i.e., g organic matter/1.724 = g organic carbon) [22].

Concentrations of major and minor ions, as well as heavy metals, for thirteen sediment samples were measured using Bench XRF Spectrometer/SPECTRO XEPOS-2006 device at the Iraqi-German Laboratory at the University of Baghdad. Samples were seived in a 2 mm seive, then powdered to 0.063 μm, and 5.0 g of each sample was used to determine the element concentrations.

Ten of heavy metals (Hg, Co, Cr, Cu, Cd, Pb, Fe, Ni, Mn, and Zn) were measured in plant and fish tissues. Dry tissue of plant and fish samples (in triplicate, each 0.2 g) were put into digestion flasks with 5 ml nitric acid (Merck) and 2 ml perchloric acid, and then heated at 90 °C until all the materials were dissolved. After digestion, the samples were diluted with deionized water to a volume of 10 ml and then filtered. The resulting solutions were analyzed using flame atomic absorption spectrophotometer [23].

### 2.4 Statistical analysis

Multivariate statistical techniques, such as Principal Component Analysis (PCA) and Agglomerative Hierarchal Cluster Analysis (AHCA), were performed using JMP 8.0 (SAS System) to determine the sources of major, minor, and heavy metals in sediment samples from Al-Hammar Marsh.

### 2.5 Pollution analysis

Pollution indices, such as Enrichment Factor (EF), are powerful tools for processing, analyzing, and conveying raw environmental information to decision makers, managers, technicians, and the public [24].

The formula to calculate EF is:

EF = (C_x_/C_y_)_S_/(C_x_/C_y_)_RS_

Where C_x_ is the measured concentration of the examined metal in the sediment sample (mg/kg), and C_y_ is concentration of immobile element in the sample (zirconium here), and (C_x_/C_y_)_RS_ is the concentration of element X to immobile element ratio in the selected reference sample [25].

In order to evaluate whether the content of a chemical element in the sediment is derived from natural or anthropogenic sources, the EF was calculated for all studied sediment samples using zirconium as the reference element. The EF is the relative abundance of a chemical element in a sediment sample compared to the bedrock. Zirconium is generally considered to mainly originate from natural lithogenic sources (rock weathering of mineral zircon), and has no significant anthropogenic source. Total elemental concentrations (ppm) in the world soil, according to [26] (Table 2), are considered to calculate EF. An EF < 2 shows deficiency to low enrichment and can be considered in the range of natural variability. 2 < EF < 5 shows low enrichment (i.e., some enrichment caused by anthropogenic input). 5 < EF < 20 is a clear indication of human influence (significant enrichment caused by anthropogenic inputs). An EF 20 to 40 represents very high enrichment and an EF > 40 represents extremely high enrichment [27, 28].

## 3 Results and discussion

### 3.1 Water analysis

High turbidity values that exceed WHO standard limits for drinking water [29] (Table 3) observed in the current study due to the high turbidity of Al-Hammar Marsh feeders (e.g., Euphrates River), as these water supplies carry large quantities of clay, silt, plankton and other microscopic organisms [30]. All TDS and TH values in water samples were considered unacceptable according to WHO standard limits for drinking water [29] (Table 3). The pH values were within the acceptable limits of WHO standards (i.e., 6.5–8.5) with the exception of St_3_, which was beyond acceptable limits in the winter season. DO levels showed a considerable decrease in summer, which is due to the poor ability of water to hold oxygen at high temperatures, as a result of higher rates of microbial metabolism [31, 32] (Table 3). On the other hand, BOD levels were found to be higher in summer than in winter (Table 3). This inverse relation between DO and BOD is expected, as high BOD levels indicate high levels of organic contaminants in water, and the microbes are working intensely to break it down, consequently consuming more oxygen and resulting in low DO levels in water [33]. All concentrations of Ca^2**+**^ for both seasons were beyond acceptable levels [29], excluding St_2_, St_6_, St_13_, and St_9_ for the summer season, which were within limits (Table 4). All Mg^2+^ concentrations were beyond the acceptable limits (WHO, 2008) except St_8_, St_11_, and St_12_ for the winter season, and St_9_ and St_13_ for summer season (Table 4). In general, all Na**^+^** concentration values for both seasons exceeded WHO limits [29] (Table 4). The concentration values of K**^+^** exceeded the allowable limits in both winter and summer seasons, except at station St_5_ in the winter season and St_9_ in summer season, which were within the allowable limits (Table 4). Cl^−^ concentrations exceeded the allowable limits in both seasons, though they were lower for summer season than winter season (Table 4). All detected values of SO_4_^2−^ exceeded the allowable limits (Table 4). The high levels of TDS, TH, and major ions (i.e., Ca^2**+**^, Mg^2+^, Na**^+^**, K**^+^**, Cl^−^, and SO_4_^2−^) in the current study can be attributed to the high salinity of Al-Hammar Marsh feeders, agriculture runoff, livestock manure (such as buffalo manure) that is widely applied in the area, domestic sewage effluents, and washing of the surrounding Sabkha during flooding from rain storms (as occurs winter and will be discussed later (Table 4)).

PO_4_^3−^, NO_3_^−^, and NO_2_^−^ concentrations were within acceptable standards limits [29]. Although these nutrients (i.e., PO_4_^3−^, NO_3_^−^, and NO_2_^−^) have relatively high concentrations at the marsh inlet area, stagnation of Al-Hammar Marsh water can increase the opportunity for plants and aquatic organisms to remove such nutrients from the water [34].

Comparison between the results from the current study and the study of [35] and [29] standard limits showed a considerable increase in concentrations of major ions (Table 4), indicating that the impact of desiccation on water quality, even after 12 years of inundation, still exists and that the marsh conditions are still departing from desirable or historical levels. The findings are consistent with other studies [e.g.,3] that noted that some water chemistry parameters of Al-Hammar Marsh, when compared with historical surveys completed before drainage [36, 37, 38, 39], revealed high increases. This considerable increase in ion concentrations is probably related to a rise in salinity in the main feeder of the marsh (e.g., the Euphrates River) and to increased flux into the water column of ions concentrated in the soil after more than a decade of drainage and evaporation [10].

Heavy metals analyses revealed ions such as Pb, Al, B, Fe, and Mn have concentrations that generally exceed Maximum Contaminant Level (MCL) standards [40] (Table 5). Analyses also revealed that all heavy metals in the current study showed an increase in concentrations at station St_1_ (marsh inlet), while nearly all these metals exhibited a distinct decrease in their concentrations at St_8_ (marsh outlet), indicating that the marsh works as a filtering sink for metals (Table 5).

In general major and minor ions, as well as heavy metals exhibit higher concentrations in winter than in summer (Tables 4, 5). Such increasing pattern in ion concentrations at most stations in the winter season was due to runoff from washing of the surrounding Sabkha during flooding by rainstorms. Additionally, the Iraqi Ministry of Water Resource orchestrates a systematic release of water into the marsh, which is usually low in winter and high in summer, resulting in increased dilution in summer and thus lower solute concentrations.

### 3.2 Sediment analysis

#### 3.2.1 Chemical analysis

Analysis results of pH showed that all sediment samples from Al-Hammar Marsh were alkaline. This is due to the high content of calcium and magnesium carbonates. In the current study, TOC% in marsh sediments is <5%, which is concordant with [41], who assumed that TOC% of <5% is mainly restricted to brackish-water lakes and marshes. Low TOC levels in this study were due to the high salinity of marsh water.

The mean concentrations of elements in Al-Hammar Marsh sediments were compared with the natural occurrences of trace elements in world soil (Table 2). Compared to [26], Al-Hammar Marsh sediments, in general, have higher mean concentrations of Ca, Mg, S, P, Cl, Sr, Cr, Ni, Zn, N, Br, Cu, Mo, and Co (Table 2). Furthermore, elements in Al-Hammar Marsh sediments were compared with the mean value of their natural abundance in Iraqi soil, according to [42]. The mean concentrations of Cr, Ni, V, Zn, Cu, and Pb in this study exceeded the mean concentrations of their background values reported by [42] (Table 2).

Applications of fertilizers, such as Nitrogen-Phosphorus-Potassium (NPK), Nitrogen-Phosphorus (NP), Monoammonium Phosphate (MAP), and Triple superphosphate (TSP) that are produced and used in Iraq may contribute to a considerable increase of some heavy metals, such as Ca, Mg, S, P, Cr, Ni, Zn, N, Cu, Mo, and Co [43], and Sr [44]. Additionally, the region of southern Iraq is well known for oil extraction activities and such activities can contribute to high sediment pollution of Pb, Cr, Cd, Co [45], Pb, V [46], and Br [47].

#### 3.2.2 Statistical analysis

a- Principal Component Analysis

PCA technique was performed by VARIMAX rotation. VARIMAX rotation was employed because orthogonal rotation minimizes the number of variables with a high loading on each component and therefore facilitates the interpretation of PCA results [48]. This technique clusters variables into groups such that variables belonging to one group are highly correlated with one another and assumes that highly correlated compounds come from the same source [49]. Eigen values in PCA indicate the significance of the components. The component with the highest Eigen value is taken to be the most significant. Eigen values should be ≥1 for proper consideration during PCA [50]. Factor loadings values of >0.75, between 0.75 and 0.5, and between 0.5 and 0.3 are classified as strong, moderate, and weak, respectively, based on their absolute values [50].

By applying PCA to the results of the chemical analyses, four principal components with Eigen values greater than 1 were extracted, which explained 94.77% of the data variation (Table 6). The first principal component PC1, which has strong factor loading of K (0.96), Zn (0.89), Ti (0.82), Fe (0.77), Ni (0.76), and moderate factor loading of Cu (0.71), Co (0.57), Se (0.69), Al (0.71), Zr (0.66) accounts for 36.07% of the variance and can be explained as anthropogenic and natural sources. Zn, Fe, Ni, Cu, Co, and Se can result from agriculture activities and wastes from oil extraction, whereas Al, K, Ti, and Zr can be derived from natural deposits. PC2, which has strong factor loadings of Mg (0.94), Cr (0.86), Mn (0.77) and Si (0.79), accounts for 28.02% of the variance. Si originates from erosion of crustal material, while Mg, Cr, and Mn can be considered of anthropogenic origin being derived from agriculture runoff from farmland. PC3, which has strong factor loading of Br (0.96), Cd (0.80), Cl (0.79), and moderate factor loading of N (0.73) and TOC% (0.58) and accounts for 15.44% of the variance, can be considered to represent anthropogenic sources. Fertilizers, human sewage and livestock manure are known to be a significant source of these elements [51]. PC4 has a strong factor loading of Pb (0.97) and moderate loading of Co (0.72), V (0.61), and As (0.60) accounts for 15.23% of the variance. Elements in PC4 have the same source, which are fertilizers and waste from oil extraction processes.

b- Cluster analysis

**Fig. 2:**
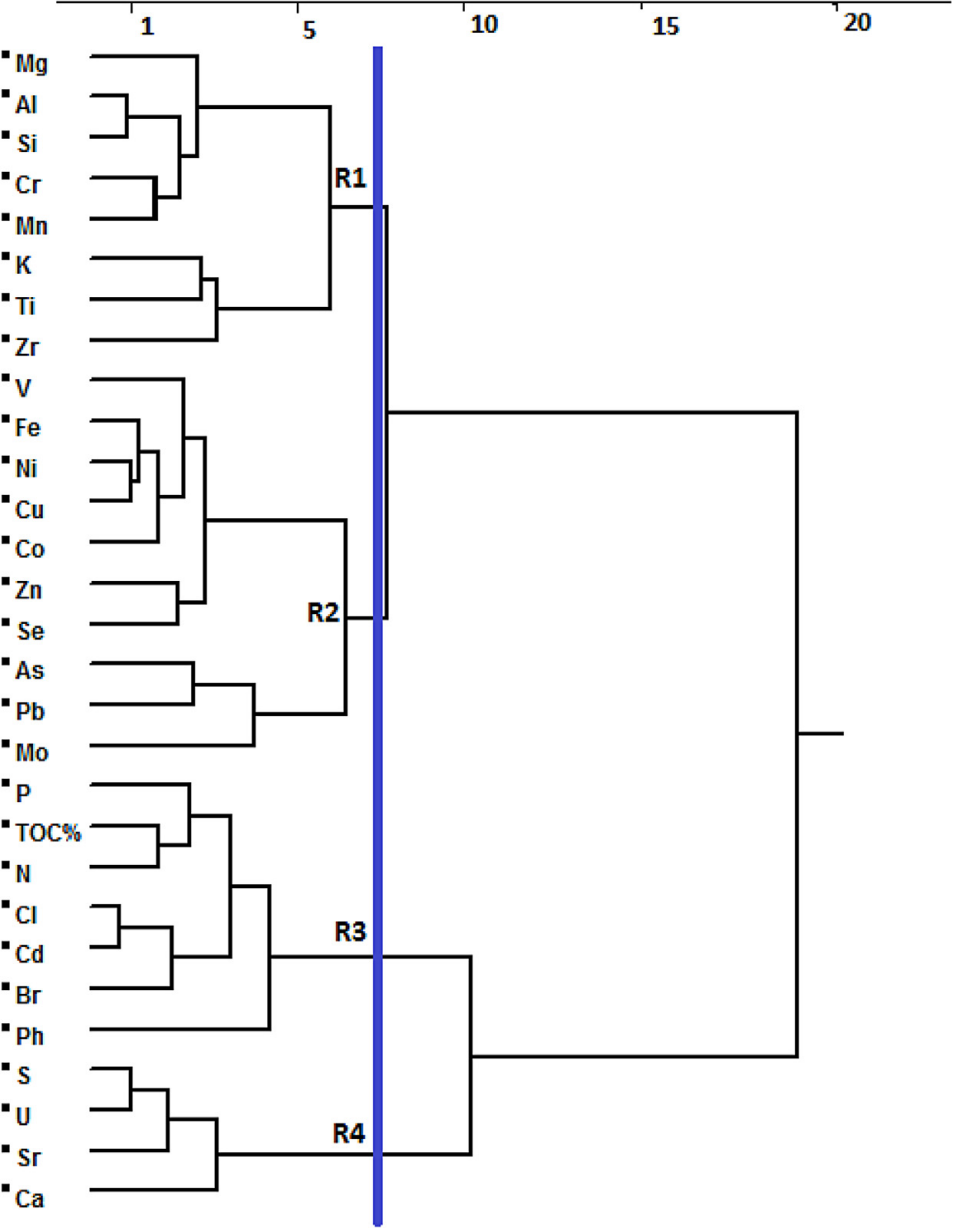
Dendrogram of elements measured and pH using Ward method.

By applying the Ward method, AHCA was performed on the results of element concentrations in sediment samples from Al-Hammar Marsh. AHCA highlighted four specific element response patterns (R1, R2, R3, and R4). The distance cluster represents the degree of association between elements, where clusters with smaller or shorter distances between them are more similar to each other than clusters with larger or longer distances between [52]. Here, cluster R2 has the shortest distance (6.98) and highest similarity to cluster R1, whereas cluster R3 is the least similar and has the greatest distance to R1 (19.33) (Fig. 2).

Elements clustering in R1 (Mg, Al, Si, Cr, Mn, K, Ti, and Zr) that dominate in the PC2 indicate natural and anthropogenic sources. Al, Si, Ti, K, and Zr are lithophile elements according to Goldschmidt’s classification of geochemical elements [53]. Lithophile elements are those showing an affinity for silicate phases and are concentrated in the silicate portion (crust and mantle) of the Earth [53]. Concentration results of Mg, Cr, and Mn show pollution of Al-Hammar Marsh sediments by these elements, which may come from fertilizers that are known to be a significant source of these elements [43, 44, 51, 54]. V, Fe, Ni, Cu, Co, Zn, Se, As, and Mo clustered in R2 (dominating in the PC1 and PC4) are indicative of anthropogenic sources (i.e., agricultural and petroleum production activities), which the Environmental Protection Agency reported as sources of contaminants [55], along with [56] who referred to some of these trace metals being released from fertilizers and from oil refineries. P, TOC%, N, Cl, Cd, and Br clustered in R3 (dominating in the PC3) can result from agricultural sources according to [57]. Nitrate-N, ammonium-N, phosphate-P, and C are the most common contaminants derived from unregulated animal waste disposal practices. These four chemicals are usually found at concentrations ranging from 1,000 to 50,000 mg/kg (elemental form) in animal wastes [58]. Cd and Br can also be from agricultural sources [44]. Elements in R4 (i.e., S, Ca, Sr, and U) are mainly of anthropogenic origin. Fertilizers can be a source for S, Ca [43, 59], Sr, and U [44].

c- Pollution analysis

**Fig. 3:**
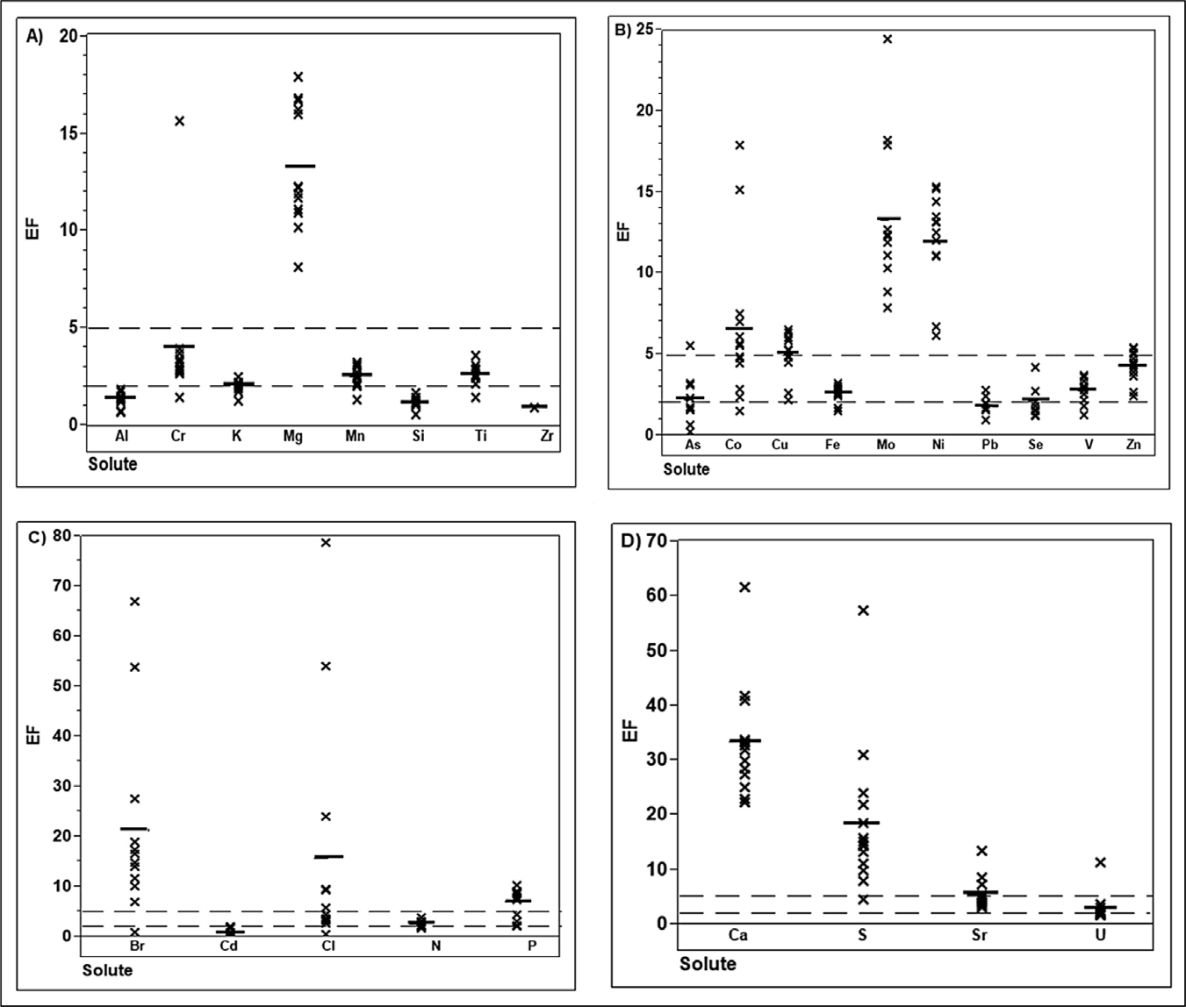
Enrichment Factor (EF) for elements. The middle horizontal thick lines represent the mean EF while the dotted horizontal thin lines represent EFs of 2 and 5. An EF of 2 is a threshold between natural and possible anthropogenic element sourcing while an EF of 5 represents a threshold between possible anthropogenic and significant anthropogenic element sourcing.

The results of EF calculations for Al-Hammar Marsh sediment samples show that EF values for S, Ca, U and Sr (clustered in R4) show a general enrichment and have mean EF values of 18.82, 33.54, 3.35 and 5.73, respectively (Fig. 3D). This enrichment indicates that anthropogenic activity is a remarkable source for these elements. P, N, Cl, Cd, and Br (clustered in R3) have mean EF values of 7.19, 2.68, 15.94, 1.00, and 21.58, respectively (Fig. 3C), and indicate a predominantly anthropogenic source. V, Fe, Ni, Cu, Co, Zn, Se, As, Pb, and Mo (clustered in R2) have mean EF values of 2.81, 2.69, 12.03, 5.01, 6.67, 4.36, 2.19, 2.25, 1.91, and 13.33, respectively (Fig. 3B), indicating the anthropogenic input of these elements in sediments in Al-Hammar Marsh. Elements Mg, Al, Si, Cr, Mn, K, and Ti, (clustered in R1) have mean EF values of 13.33, 1.45, 1.27, 4.10, 2.55, 2.13, and 2.67, respectively (Fig. 3A), indicating that sediments are significantly polluted by Mg and Mn, minimally polluted by Cr, and not polluted by Al, K, Ti and Si. It is worth mentioning that this pollution analysis is in good agreement with both AHCA and PCA analyses in determining the elemental sourcing (i.e., natural and/or anthropogenic).

### 3.3 Plant analysis

**Fig. 4:**
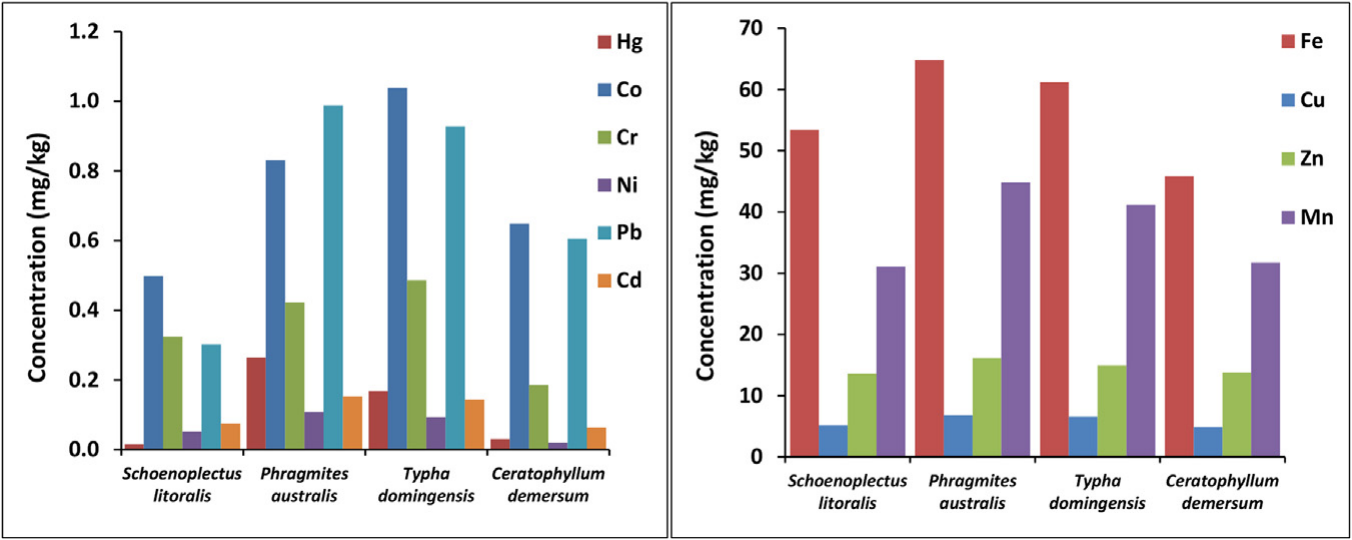
Distribution of heavy metals in plant sample.

In the current study, investigation of plant pollution by studying heavy metals content in plant tissue provides useful information on the status of Al-Hammar Marsh environment. Pollution for four plant species of *Schoenoplectus litoralis*, *Phragmites australis, Typha domingensis,* and *Ceratophyllum demersum* was investigated by examining ten heavy metals (Hg, Co, Cr, Ni, Pb, Cd, Cu, Zn, Mn, and Fe). Results of plant analysis show that the mean concentration of heavy metals are in the order of Fe > Mn > Zn > Cu > Co > Pb > Cr > Hg > Cd > Ni (Table 7 and Fig. 4), and all heavy metals detected in plant samples were much greater than those detected in water samples at same sampling stations; however, all were below the permissible limits.

### 3.4 Fish analysis

**Fig. 5:**
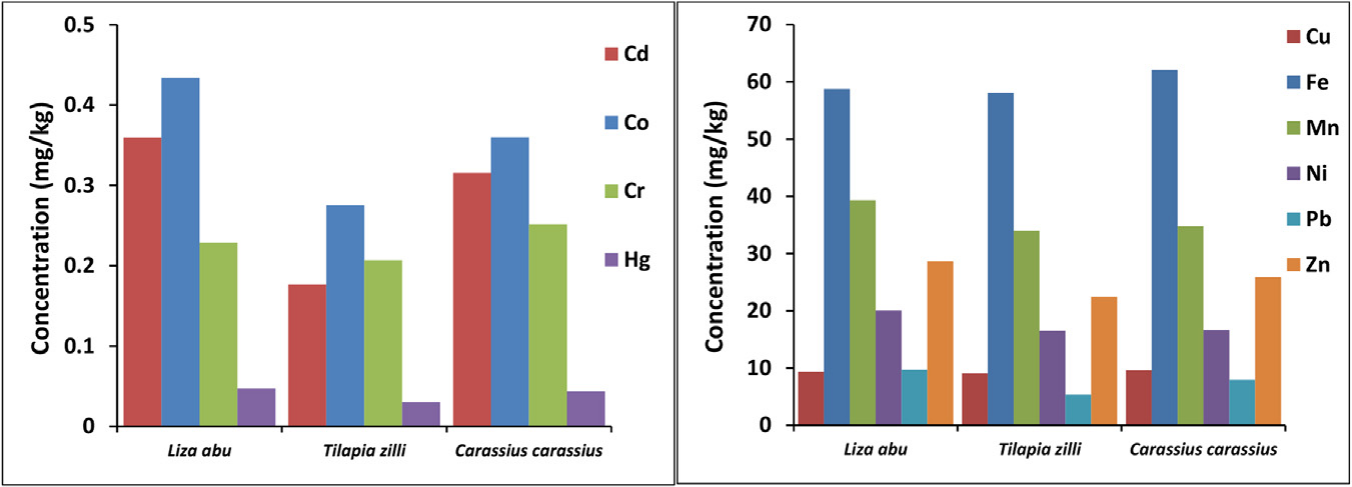
Distribution of heavy metals in fish samples.

Fish are often used to study their body burdens and the transfer of pollutants in the food web [61]. They can be good indicators of the bioaccumulation resulting from the contamination of the environment [61]. In the present study, fifteen fish samples of three fish species were analyzed for their heavy metals content. This study was carried out to evaluate the effect of water and sediment pollution on fish living in Al-Hammar Marsh water. Results show that mean concentrations of heavy metals was in the order of Fe > Mn > Zn > Ni > Cu > Pb > Co > Cd > Cr > Hg (Table 8 and Fig. 5), and the concentrations of heavy metals were several times higher than their concentrations in water samples; this is a clear indication of bioaccumulation of heavy metals in fish tissues. It appears that Co, Cr, Cu, Fe, and Hg concentrate in *Carassius carassius* more than *Tilapia zilli* and *Liza Abu*, while Cd, Mn, Ni, Pb and Zn concentrate in *Liza Abu* more than *Carassius carassius, and Tilapia zilli*. The heavy metals analysis of fish samples shows that Cd and Co levels exceeded permissible limits in some fish samples, while Mn, Ni, and Pb concentrations were above the permissible limits in all fish species (Table 8).

## 4 Conclusions

1- Decreasing of Tigris and Euphrates discharges during the past decades due to drought conditions and upstream damming, as well as the increasing stress of wastewater effluents from agricultural, residential, and industrial (mainly oil extraction) activities, led to degradation of the downstream Al-Hammar Marsh water quality in terms of physical, chemical, and biological properties. As such properties were found to consistently exceed the historical objectives as well as WHO objectives.
2- The Marsh works as a natural filtration and bioremediation system, as nearly all observed major ions and heavy metals in water showed a distinct decreasing trend at the marsh outlet station compared to other stations.
3- The applied multivariate statistical techniques, such as PCA and AHCA, identified the possible sources of contaminants in sediments: some solutes are of anthropogenic sources (mainly fertilizers and petroleum extraction wastes), and others are from natural sources. Moreover, EF analysis which was used along with PCA and AHCA to support the element sourcing gave results compatible with PCA and AHCA findings.
4- Heavy metals detected in plant species were within acceptable limits, however, heavy metal concentrations in fish samples showed that some fish samples were contaminated by Cd and Co, and all of them were contaminated by Pb, Mn, and Ni. This is a clear indication of bioaccumulation and biomagnification of heavy metals in fish tissues.

## Declarations

### Author contribution statement

H.F.A. Al-Gburi: Conceived and designed the experiments; Performed the experiments; Analyzed and interpreted the data; Contributed reagents, materials, analysis tools or data; Wrote the paper.

B.S. Al-Tawash, H.S. Al-Lafta: Analyzed and interpreted the data; Contributed reagents, materials, analysis tools or data; Wrote the paper.

### Funding statement

This research did not receive any specific grant from funding agencies in the public, commercial, or not-for-profit sectors.

### Competing interest statement

The authors declare no conflict of interest.

### Additional information

No additional information is available for this paper.

## Figures and Tables

**Table 1:** Locations of the water, sediment, plants, and fish samples that were collected from Al-Hammar Marsh.

Station No.	Water samples	Sediment samples	Plant samples	Fish samples	Coordinates		Site name	Province
					N	E		
1	St_1_	St_1_	P_1_	F_1_	30°53'49.38”	46°29'47.87”	Suk Al-Shuyukh	Thiqar
2	St_2_	St_2_	P_2_	F_2_	30°48'5.24”	46°35'3.87”	Al-Sinaf	Thiqar
3	St_3_	St_3_	P_3_	F_3_	30°50'42.55”	46°58'37.14”	Hor Abu tina	Thiqar
4	St_4_	St_4_	P_4_	F_4_	30°39'20.99”	47°38'25.15”	Naggarah	Basra
5	St_5_	St_5_	P_5_	F_5_	30°56'48.73”	46°46'1.99”	Al-Chibayish	Thiqar
6	St_6_	St_6_	P_6_		30°40'29.96”	47°28'25.99”	Shilaychiya	Basra
7	St_7_	St_7_	P_7_		30°35'43.87”	47°41'51.25”	Qarmat Ali	Basra
8	St_8_		P_8_	F_8_	30°34'43.61”	47°44'16.73”	Qarmat Ali	Basra
9	St_9_	St_9_	P_9_	F_9_	30°49'26.51”	47°29'46.61”	Al-Shafi	Basra
10	St_10_	St_10_	P_10_		30°38'39.11”	47°41'6.39”	Al-Mashab	Basra
11	St_11_	St_11_			30°49'8.03”	46°37'1.36”	Kirmashia	Thiqar
12	St_12_	St_12_			30°42'6.99”	47°35'3.43”	Al-Barga	Basra
13	St_13_				30°50'44.87”	46°43'9.48”	Al-Bithij	Thiqar
14		*S_1_			30°38'20.83”	47°40'40.87”	Al-Hartha	Basra
15		*S_2_			30°38'24.79”	47°42'35.32”	Al-Hartha	Basra
16		*S_3_			30°41'0.66”	47° 7'27.70”	Rumillah oil field	Basra
17		*S_3A_			30°39'26.59”	47°35'32.93”	Al-Hartha	Basra
18		*S_3B_			30°40'44.72”	47°36'24.34”	Al-Hartha	Basra
19		*S_6_			30°36'43.88”	47°40'1.31”	Al-Hartha	Basra

* Dry sediments.

**Table 2:** Results of chemical analysis (in mg/kg) of sediments for the winter season in Al-Hammar Marsh.

Station	pH	OM%	TOC%	Ca	Si	Fe	Mg	S	Al	K	Ti	P	CI	Sr	Mn	Cr	Ni	V	Zr	Zn	N	Br	Cu	Pb	Mo	As	U	Co	Se	Cd	Hg
**St_1_**	7.69	4.59	2.66	**201190**	99751	27884	**26888**	**19584**	12618	6981	3395	**1995**	**1883**	**977**	650	191	***137***	*86*	71	***60***	**36.0**	**32.90**	***28.04***	*11.63*	**11.60**	3.79	***3.60***	3.00	**0.2**	0.1	<1
**St_2_**	7.72	4.05	2.35	**135311**	191445	37705	**41592**	**5302**	52591	10535	4428	**828**	**1155**	**520**	**1029**	***300***	***189***	***117***	117	***78***	**32.0**	**34.30**	***39.22***	*11.88*	**9.80**	4.73	*0.90*	**23.75**	**0.2**	0.04	<1
**St_3_**	7.60	4.56	2.65	**164403**	133146	36229	**27593**	**2599**	35115	10652	4435	**2255**	**2932**	**471**	741	***259***	***166***	***107***	112	***78***	**35.0**	**28.20**	***34.43***	*10.30*	**6.60**	3.29	*0.90*	**16.83**	**0.2**	0.07	<1
**St_5_**	7.50	4.62	2.68	**173052**	130762	**38153**	**22786**	**5647**	35354	8999	3958	**2269**	**1150**	**804**	843	174	***185***	***101***	90	***83***	**34.0**	**21.50**	***39.78***	*12.63*	**11.00**	3.60	*1.10*	**36.57**	**0.2**	0.04	<1
**St_7_**	7.45	4.13	2.39	**178914**	131043	33655	**24517**	**4017**	33353	9664	**5324**	**2245**	**973**	**717**	694	**211**	***160***	***100***	95	***76***	**30.0**	**18.90**	***31.95***	*10.95*	**7.60**	2.88	*0.87*	**12.27**	**0.2**	0.09	<1
**St_9_**	7.73	4.35	2.52	**126948**	182842	**43099**	**41001**	**5250**	51082	9988	4110	**1098**	**1294**	**505**	**963**	***302***	***179***	***115***	120	***75***	**28.0**	**33.80**	***39.54***	*10.68*	**14.40**	4.81	***1.90***	**19.74**	**0.3**	0.04	<1
**St_10_**	7.86	4.17	2.42	**166476**	168537	35432	**37744**	**1465**	46610	11025	4219	**661**	**1782**	**420**	706	**216**	***160***	*92*	107	***79***	**24.0**	**12.80**	***34.99***	*10.95*	**8.90**	3.26	*1.10*	**20.13**	**0.4**	0.08	<1
**St_11_**	7.57	4.63	2.68	**133024**	145629	**43938**	**24427**	**4341**	39894	10610	4417	**2564**	**833**	**543**	840	**214**	***215***	***134***	105	***91***	**31.0**	**30.90**	***45.21***	*12.72*	**9.00**	**5.79**	*1.30*	**50.65**	**0.6**	0.06	<1
**St_12_**	7.56	4.68	2.71	**162759**	135203	36719	**24916**	**3426**	34988	10809	4154	**2314**	**1385**	**513**	760	**216**	***186***	66	106	***81***	**29.0**	**18.10**	***43.22***	*10.68*	**7.90**	0.46	*1.00*	**12.66**	**0.4**	0.05	<1
*S_3_	7.90	5.30	3.07	**134239**	170126	20077	**19186**	**14573**	29304	9124	4085	**2198**	**188**	**454**	527	***934***	**74**	67	89	41	**35.0**	1.70	13.26	*8.17*	**7.10**	2.42	<0.10	7.00	<0.5	0.24	<1
*S_2_	8.07	5.10	2.96	**175482**	120664	34026	**29457**	**6576**	31966	10278	4019	**2161**	**11180**	**588**	682	174	***154***	*78*	171	***72***	**38.0**	**54.89**	***30.92***	*10.12*	**9.00**	2.12	<1	**11.17**	<0.5	0.18	<1
*S_6_	7.69	4.65	2.70	**136026**	133427	37566	**33221**	**6788**	35999	11465	4092	**2409**	**21180**	**462**	702	180	***178***	*100*	98	***89***	**40.0**	**88.60**	***35.15***	*11.23*	**6.80**	3.03	*1.10*	**15.10**	<0.5	0.29	<1
*S_3B_	7.72	4.95	2.87	**137598**	133520	**38846**	**27093**	**3007**	35496	10793	4169	**2282**	**15460**	**447**	637	200	***185***	*91*	104	***79***	**41.0**	**117.20**	***36.83***	*11.79*	**8.60**	**9.80**	<1	**13.76**	<0.5	0.26	<1

**Mean**	7.70	4.60	2.67	155802	144315	35641	29263	6352	36490	10071	4216	1945	4723	571	752	275	167	96	107	76	33	37.98	34.81	11.06	9.10	3.84	1.38	18.66	0.30	0.12	

**SD**	0.17	0.37	0.21	23227	26313	6188	7064	5107	10184	1175	427	637	6745	165	137	203	34	20	23	13	5	32.03	8.08	1.19	2.20	2.23	0.84	12.67	0.14	0.09	

*[26]				13700	330000	38000	6300	850	71300	13600	4600	800	450	300	850	200	40	100	300	50	20	5	20	17	2	5	1.8	8	0.4	0.35	<1

**[42]																238	91	71		54			16	6			0.2				

Bold values represent concentrations that exceed [26] values.

Italic values represent concentrations that exceed [42] values.

* Elements distribution limits in world soil according to [26].

** Background mean values of trace elements in Mesopotamia soil and sediments according to [42].

**Table 3:** Physiochemical parameters for water samples in Al-Hammar Marsh for winter (W) and summer (S) seasons.

Station No.	Water depth (m)		Turbidity (NTU)		TDS (mg/l)		EC (dS/cm)		TH (mg/l)		pH		DO (mg/l)		BOD		COD (mg/l)	
	W	S	W	S	W	S	W	S	W	S	W	S	W	S	W	S	W	S
St_1_	2.5	2.5	17.65	78.4	13602	10298	17.5	11.1	6353	2600	7.90	8.30	8.30	1.50	8.30	30.0	78	90
St_2_	2	1.2	9.88	29.2	6834	2384	7.9	3.5	1950	970	7.65	8.10	8.33	1.90	7.60	13.0	61	18
St_3_	2	1.5	45.91	11.3	9980	4000	16.7	6.1	3300	1668	9.00	8.10	8.85	0.34	8.17	1.6	106	40
St_4_	2	1.2	18.80	54.0	7140	6890	11.3	3.3	2450	2231	8.37	8.10	8.60	7.80	6.45	3.1	104	30
St_5_	1.5	1.0	10.90	28.0	5732	3142	6.6	4.0	1700	1076	8.25	8.00	8.81	4.10	6.46	5.0	44	32
St_6_	>4	1.0	10.31	34.0	5532	3076	7.3	4.0	950	1241	8.31	8.00	8.88	0.02	6.48	10.0	66	41
St_7_	>4	NM	6.10	NM	3090	NM	4.6	NM	1100	NM	8.36	NM	8.95	NM	6.60	NM	55	NM
St_8_	>4	>4	5.63	13.0	2744	3068	4.0	4.3	900	1164	8.27	8.30	8.30	3.00	5.21	5.5	59	32
St_9_	1.5	1.2	11.70	99.1	9666	1698	11.2	2.3	2200	728	8.30	8.30	7.70	5.20	6.16	2.5	78	42
St_10_	>4	NM	10.50	NM	7646	NM	9.6	NM	2000	NM	8.26	NM	8.43	NM	7.80	NM	84	NM
St_11_	2.5	NM	10.80	NM	3963	NM	6.2	NM	2010	NM	7.67	NM	4.69	NM	NM	NM	48	NM
St_12_	>4	NM	13.50	NM	6170	NM	9.3	NM	3320	NM	8.57	NM	8.98	NM	NM	NM	96	NM
St_13_	NM	4.0	NM	15.4	NM	4000	NM	6.3	NM	679	NM	8.00	NM	3.10	NM	1.6	NM	37

SD		0.98	10.22	29.2	2983	2531	4.11	2.5	1424	627	0.36	0.13	1.13	2.32	0.95	8.59	21	19

Mean		1.6	14.3	40.3	6841	4284	9.3	4.9	2352	1373	8.28	8.09	8.23	2.99	6.51	8.0	73	40

WHO (2008)			5		1000		250		500		6.5–8.5						4	

NM: Not Measured.

**Table 4:** Ions concentrations (mg/l) in Al-Hammar Marsh water for two sampling seasons for current study, mean concentrations of these ions for a previous study by [35], and [29] standard limits.

Station	Ca^2+^		Mg^2+^		Na^+^		K^+^		Cl^−^		HCO_3_^−^		SO_4_^2−^		PO_4_^3−^		NO_3_^−^		NO_2_^−^	
	W	S	W	S	W	S	W	S	W	S	W	S	W	S	W	S	W	S	W	S
St_1_	***655***	***460***	***920***	***348***	***2300***	***1520***	***78***	***45***	***4050***	***3122***	*243*	*224*	***3700***	***1800***	0.28	0.15	9.1	3.1	0.11	0.09
St_2_	***320***	***150***	***276***	***137***	***930***	***325***	***25***	***15***	***1622***	***675***	*307*	*174*	***1750***	***800***	0.12	0.28	1.6	2.5	0.09	0.10
St_3_	***460***	***200***	***510***	***270***	***2170***	***560***	***55***	***18***	***2890***	***1120***	*222*	*235*	***3000***	***1250***	0.15	0.18	5.3	3.3	0.10	0.10
St_4_	***360***	***254***	***372***	***369***	***1875***	***1390***	***50***	***43***	***2850***	***2587***	*217*	*130*	***1440***	***1500***	0.15	0.21	3.9	4.2	0.10	0.10
St_5_	***220***	***169***	***276***	***151***	***1533***	***605***	*12*	***19***	***2375***	***1035***	*266*	*143*	***1800***	***760***	0.12	0.30	3.3	4.7	0.10	0.10
St_6_	***240***	***146***	***204***	***202***	***992***	***565***	***26***	***21***	***1662***	***1021***	*251*	*172*	***1322***	***900***	0.15	0.34	3.5	3.7	0.09	0.13
St_7_	***200***		***144***		***496***		***15***		***860***		*259*		***875***		0.21		3.8		0.09	
St_8_	***180***	***177***	*108*	***166***	***488***	***635***	***13***	***23***	***665***	***1030***	*254*	*121*	***910***	***900***	0.18	0.34	4.4	4.0	0.09	0.10
St_9_	***320***	*96*	***336***	*113*	***1695***	**280**	***45***	*11*	***2375***	**405**	*231*	*139*	***2800***	***500***	0.18	0.28	3.8	2.3	0.09	0.13
St_10_	***300***		***300***		***1373***		***38***		***2137***		*246*		***2300***		0.15		3.4		0.10	
St_11_	***240***		99		***956***		***66***		***1330***		*232*		***967***		**0.55**		0.4		0.12	
St_12_	***270***		90		***1433***		***74***		***2230***		*272*		***1200***		**0.7**		0.3		0.09	
St_13_		*119*		87		***760***		***26***		***1105***		*122*		***865***		0.28		2.7		0.10

SD	132	108	231	101	598	434	23	11.9	942	897	24	42	921	407	0.19	0.07	2.33	0.82	0.01	0.01

Mean	314	197	303	205	1353	738	41	24.4	2087	1344	250	162	1839	1031	0.25	0.26	3.6	3.4	0.10	0.11

[35]	89		100		289		7		487		64		247							
[29]	100		125		200		12		250				250		0.4		50		3	

**Table 5:** Heavy metal concentrations (in μg/l) in water samples for winter (W) and summer (S) seasons for Al-Hammar Marsh.

Station	As		Cd		Cr		Cu		Hg		Pb		Se		Zn		Al		B		Be		Co		Fe		Li		Mn		Mo		Ni		U		V		Sr	
	W	S	W	S	W	S	W	S	W	S	W	S	W	S	W	S	W	S	W	S	W	S	W	S	W	S	W	S	W	S	W	S	W	S	W	S	W	S	W	S
St_1_	**20**	**20**	1.3	0.1	58	8	30.2	36.9	**2.8**	<0.2	**16.4**	3.5	30	30	167	28	**240**	**700**	**>1000**	**>1000**	0.3	<0.3	1.8	2.3	**1750**	**990**	60	120	26	**651**	36	29	37.9	19.5	11.7	9.47	9	22	>1000	>1000
St_2_	10	**10**	0.6	<0.1	22	3	31.4	9.5	1	<0.2	**43.1**	0.5	10	10	132	36	**800**	70	**>1000**	**850**	<0.3	<0.3	3.5	0.2	**1520**	90	50	30	**68.9**	**111**	41	11	37.3	5.4	16.4	4.1	19	8	>1000	>1000
St_3_	10	<10	0.1	<0.1	5	23	18.7	7.3	0.3	0.3	9.8	0.8	20	10	64	53	100	140	**>1000**	**>1000**	<0.3	<0.3	2	0.3	**2190**	**330**	110	50	43.8	**53.1**	28	10	37.9	12.6	6.38	1.13	6	2	>1000	>1000
St_4_	10	<10	0.1	<0.1	6	10	22.3	8.1	<0.2	<0.2	**23.4**	1.9	10	20	88	31	**1390**	**480**	**>1000**	**>1000**	2.4	<0.3	3.1	0.8	**3610**	**650**	90	70	47.4	30.6	25	13	46.5	10.7	5.17	4.11	32	5	>1000	>1000
St_5_	10	<10	0.2	<0.1	5	<1	46.4	2.1	<0.2	<0.2	**50.5**	0.4	10	10	189	8	**600**	**230**	**>1000**	**920**	<0.3	<0.3	3.8	0.1	**2040**	<20	60	30	45.6	10.5	35	13	47.7	2.8	7.83	4.51	14	8	>1000	>1000
St_6_	10	10	<0.1	0.1	6	12	36.2	4.7	<0.2	0.3	**48.6**	0.7	10	10	145	47	**340**	80	**1000**	**870**	<0.3	<0.3	3.2	0.3	**1700**	180	60	40	**56.6**	8.7	22	10	37.1	11.1	3.88	2.34	8	9	>1000	>1000
St_7_	<10		<0.1		4		18.6		0.2		**17.8**		10		68		160		**610**		<0.3		2		**920**		40		20.7		11		31.9		2.67		7		>1000	
St_8_	<10	<10	<0.1	<0.1	5	7	6.2	6.2	0.2	<0.2	<0.2	1.4	<10	10	27	36	<50	<50	470	**600**	<0.3	<0.3	0.5	0.3	**640**	240	40	40	8	42.1	10	7	25.5	5.7	2.53	2.12	6	5	>1000	>1000
St_9_	10	<10	0.3	<0.1	10	20	70.6	5.4	<0.2	0.3	**71.9**	1	20	<10	168	86	**420**	110	**>1000**	390	<0.3	<0.3	4.2	0.4	**5440**	250	80	40	**96**	16.2	40	10	49.6	13	5.2	1.65	8	7	>1000	>1000
St_13_	10	10	0.1	<0.1	6	2	35.7	8.3	0.3	<0.2	**23.6**	0.4	10	10	439	16	**230**	60	**>1000**	>1000	<0.3	<0.3	2.7	0.2	**1270**	60	240	40	32.2	149	18	12	32.6	3.6	4.82	2.45	7	6	>1000	>1000

Mean					12.7	10.6	31.6	9.8			33.9	1.17	14.4	14.3	149	38	475	233					2.7	0.5	2108	348	83	51	44.5	119	26.6	12.8	38	9.4	6.65	3.54	11.6	8	>1000	>1000

SD					16.8	7.5	17.8	10.4			20.6	1	7.3	7.9	115	22.8	408	233					1.1	0.7	1425	316	59	28	25	205	11.3	6.4	7.6	5.4	4.3	2.5	8.3	5.7		

MCL	10		5		100		1300		2		15		50		5000		50–200		500		4		50		300		700		50		70		70		30		9		4000	

Bold values represent concentrations that exceed MCL.

**Table 6:** PCA loadings of major and trace elements on varimax rotated principal components.

Element	Component			
	1	2	3	4
**Mg**	0.089	0.926*	0.350	–0.655
**Al**	0.655*	0.747	0.098	–0.089
**Si**	0.551	0.791*	–0.209	0.153
**P**	–0.091	–0.939	0.260	0.116
**S**	–0.972	–0.172	0.083	–0.047
**CI**	0.118	–0.463	0.797*	–0.296
**K**	0.970*	0.124	0.186	–0.038
**Ca**	–0.756	–0.510	–0.204	–0.342
**V**	0.434	0.561	–0.217	0.610*
**Cd**	–0.003	–0.513	0.802*	–0.278
**Cr**	0.345	0.861*	–0.311	0.063
**Mn**	0.350	0.772*	–0.361	0.348
**Fe**	0.771*	0.399	–0.023	0.481
**Co**	0.576*	0.184	–0.308	0.720*
**Ni**	0.762*	0.203	–0.056	0.603
**Cu**	0.716*	0.284	–0.246	0.561
**Zn**	0.897*	–0.059	0.026	0.420
**As**	0.083	0.325	0.664	0.602*
**Se**	0.700*	–0.020	–0.176	0.498
**Br**	0.052	–0.192	0.967*	–0.012
**Sr**	–0.923	–0.294	–0.199	0.064
**Mo**	–0.533	0.070	–0.215	0.283
**Pb**	–0.089	0.007	–0.058	0.976*
**U**	–0.975	–0.060	0.109	0.027
**Ti**	0.820*	0.099	–0.334	–0.021
**Zr**	0.661*	0.269	0.225	–0.432
**pH**	–0.008	–0.911	0.064	–0.310
**TOC%**	–0.209	–0.741	0.582*	–0.053
**N**	–0.318	–0.557	0.730*	–0.122
**Variance explained by component %**	36.074	28.022	15.435	15.234
**Cumulative variance explained by component %**	36.074	64.096	79.531	94.765

* Significant variable.

**Table 7:** Heavy metal concentrations (mg/kg) in plant samples with critical concentrations of trace metals in plant tissues.

Station No.	Plant species	Hg	Co	Cr	Fe	Ni	Pb	Cd	Cu	Zn	Mn
P_1_	*Schoenoplectus litoralis*	0.014	0.506	0.329	55.41	0.051	0.325	0.075	5.217	14.10	30.56
P_6_	*Schoenoplectus litoralis*	0.010	0.425	0.350	50.68	0.057	0.209	0.081	4.845	13.56	28.91
P_10_	*Schoenoplectus litoralis*	0.019	0.563	0.293	54.20	0.046	0.371	0.070	5.461	13.09	33.74
P_2_	*Phragmites australis*	0.261	0.869	0.415	61.72	0.105	1.021	0.094	6.821	15.90	48.02
P_7_	*Phragmites australis*	0.300	0.728	0.468	68.10	0.096	0.902	0.210	7.069	16.65	41.79
P_8_	*Phragmites australis*	0.232	0.895	0.386	64.63	0.124	1.040	0.155	6.598	15.78	44.61
P_3_	*Typha domingensis*	0.190	1.021	0.501	63.23	0.092	1.036	0.134	6.715	14.91	43.16
P_4_	*Typha domingensis*	0.210	0.925	0.465	58.60	0.079	0.811	0.097	5.966	14.46	38.97
P_9_	*Typha domingensis*	0.105	1.169	0.492	61.74	0.110	0.935	0.201	7.011	15.38	41.50
P_2_	*Ceratophyllum demersum*	0.062	0.723	0.213	48.15	0.031	0.431	0.054	5.353	14.34	25.34
P_5_	*Ceratophyllum demersum*	0.011	0.641	0.165	43.33	0.011	0.729	0.072	4.503	13.81	36.22
P_7_	*Ceratophyllum demersum*	0.017	0.583	0.180	46.01	0.018	0.656	0.064	4.764	13.25	33.65

Mean		0.119	0.754	0.354	56.31	0.068	0.705	0.108	5.860	14.602	37.20
SD		0.11	0.22	0.12	7.97	0.04	0.30	0.05	0.95	1.13	6.94
Critical concentrations in plants		*0.5–1	*10–20	*1–2	*300–600	*20–30	**30–300	*5–10	*15–20	*150–200	**400–1000

* [60].

** [44].

**Table 8:** Heavy metals concentration (mg/kg) in fish species in Al-Hammar Marsh water with the Maximum Permitted Concentration (MPC). Bold values represent concentrations that exceed MPC.

Station No.	Fish species	Cd	Co	Cr	Cu	Fe	Hg	Mn	Ni	Pb	Zn
F_1_	*Liza abu*	0.126	**0.365**	0.202	7.96	55.63	0.039	**36.45**	**22.31**	**6.31**	26.30
F_4_	*Liza abu*	0.146	**0.418**	0.193	8.47	56.52	0.046	**43.45**	**14.41**	**8.21**	25.78
F_3_	*Liza abu*	**0.506**	**0.396**	0.186	10.72	60.51	0.048	**38.40**	**21.10**	**10.86**	32.21
F_5_	*Liza abu*	**0.825**	**0.508**	0.352	9.83	63.73	0.063	**40.12**	**29.20**	**13.45**	27.46
F_8_	*Liza abu*	0.195	**0.482**	0.211	9.82	57.36	0.039	**38.10**	**13.14**	**9.51**	31.51
F_1_	*Tilapia zilli*	0.080	0.223	0.150	8.87	53.24	0.021	**30.02**	**11.41**	**1.95**	21.21
F_2_	*Tilapia zilli*	0.021	0.195	0.141	7.21	56.62	0.016	**34.16**	**14.12**	**2.38**	17.56
F_3_	*Tilapia zilli*	**0.698**	**0.511**	0.346	10.98	65.36	0.058	**41.23**	**28.39**	**14.74**	31.10
F_5_	*Tilapia zilli*	0.014	**0.262**	0.185	8.11	57.10	0.034	**33.20**	**13.46**	**4.28**	18.65
F_8_	*Tilapia zilli*	0.071	0.185	0.212	10.09	58.21	0.023	**31.41**	**15.12**	**3.34**	23.82
F_3_	*Carassius carassius*	0.463	**0.431**	0.206	11.57	62.20	0.052	**33.30**	**16.13**	**6.45**	30.04
F_4_	*Carassius carassius*	0.036	0.224	0.206	9.43	55.75	0.030	**32.36**	**11.39**	**0.09**	20.13
F_5_	*Carassius carassius*	0.246	**0.471**	0.282	7.94	62.13	0.045	**35.11**	**18.26**	**11.56**	28.32
F_8_	*Carassius carassius*	**0.767**	**0.506**	0.391	11.21	69.05	0.065	**37.41**	**25.15**	**16.38**	29.66
F_9_	*Carassius carassius*	0.065	0.167	0.173	7.88	61.34	0.026	**35.60**	**12.33**	**5.23**	21.09

Mean		0.286	0.358	0.220	9.40	59.62	0.040	35.97	17.83	7.64	25.92

SD		0.29	0.13	0.080	1.39	4.30	0.02	3.80	6.06	4.97	4.94

MPC		*0.5	**0.04–0.26	*1	*30	**18.5–72.3	*0.5	***5	**0.01–2.78	*0.5	*150

* [62].

** [63].

*** [64].
